# Research on the Filtering Algorithm in Speed and Position Detection of Maglev Trains

**DOI:** 10.3390/s110707204

**Published:** 2011-07-14

**Authors:** Chunhui Dai, Zhiqiang Long, Yunde Xie, Song Xue

**Affiliations:** College of Mechatronics Engineering and Automation, National University of Defense Technology, Changsha, Hunan, China; E-Mails: daichunhui1984@hotmail.com (C.D.); chaorendai@sina.com (Y.X.); songself@126.com (S.X.)

**Keywords:** permanent magnet electrodynamic suspension train, position sensor, speed and position detection, track-differentiator, filtering character

## Abstract

This paper introduces in brief the traction system of a permanent magnet electrodynamic suspension (EDS) train. The synchronous traction mode based on long stators and track cable is described. A speed and position detection system is recommended. It is installed on board and is used as the feedback end. Restricted by the maglev train’s structure, the permanent magnet electrodynamic suspension (EDS) train uses the non-contact method to detect its position. Because of the shake and the track joints, the position signal sent by the position sensor is always aberrant and noisy. To solve this problem, a linear discrete track-differentiator filtering algorithm is proposed. The filtering characters of the track-differentiator (TD) and track-differentiator group are analyzed. The four series of TD are used in the signal processing unit. The result shows that the track-differentiator could have a good effect and make the traction system run normally.

## 1. Introduction

**Figure 1.**
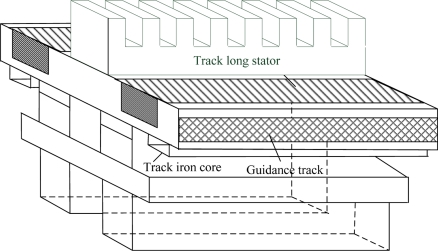
The long stator and track structure.

A permanent magnet electrodynamic suspension (EDS) train uses a linear motion actuator to achieve traction [1]. Its current transformer is placed on the ground. The long (primary) stators of the linear synchronous motor are laid on the middle of the tracks. The permanent magnet for traction is installed at the bottom of the vehicle which faces the long stators. This is shown in Figure 1. This kind of traction mode has high power factor and high efficiency the characteristics, allowing it to attain high acceleration and speed. The coils of the long stators can produce a travelling wave magnetic field. To make the traveling magnetic field synchronize with the magnetic field produced by the permanent magnet, the traction system needs to detect the position of the secondary, only so the current can be modulated to control the output force. Then the detection of the secondary position is extremely important. The error detection will not only make the vehicle run abnormally, but also cause serious damage to the correlated equipments [2–4].

**Figure 2.**
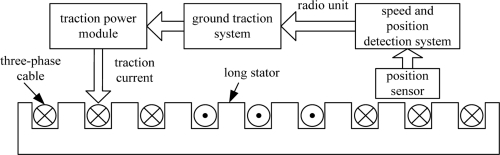
Composition of the synchronous traction system.

The synchronous traction system of a permanent magnet EDS train comprises the speed and position detection system, the radio unit, ground traction system and traction power module. It is shown in the Figure 2. In the synchronous traction system, there are two methods to detect position of the motor’s secondary. One method is to detect the counter electromotive force. The other is achieved by the position sensors. The former one is called sensorless. When the train runs at a very low speed, the sensorless method cannot work very well, because of the weak counter electromotive force and strong noise. During the slow running period, the position of the train is determined by sensors. This kind of method is also used in electric rotating motors. The electric rotating motor uses a photoelectric encoder, rotary transformer or inductive rotation sensor to detect position or speed. The character of the inductive rotation sensor is that the distance between detection probe and speed gear is non-contact and unchanged. But the same usage in a permanent magnet EDS train has its own special problems. The position sensor is a kind of on-board equipment. Its detection gap fluctuates with the levitation gap. In addition, its faced stack long stators do not have a continuous conductor surface, because the long track stators are spliced with joints of different sizes. Also the sensors are easily affected by all kinds of magnetic field, including the traveling magnetic field and the suspension magnetic field. These problems are different from the speed detection of the electric rotating motor, so this paper examines the sensors’ signal processing, dealing especially with error signal.

## 2. Principle of Speed and Position Measurement Based on the Long Stators

**Figure 3.**
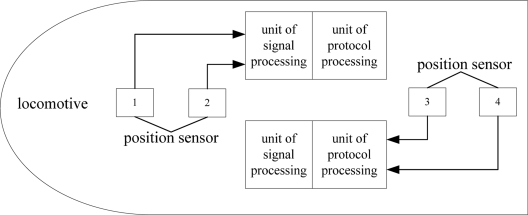
Structure of the speed and position detection system.

For a maglev train, the speed and position detection system is necessary and crucial [5]. It carries out the task of detecting the speed and position of the train. The operation control system also takes advantage of it to achieve centralized control. In order to make the speed and position detection system reliable and safe, a convenient structure is needed [6,7]. This is shown as Figure 3.

The position sensor’s detection object is the long stator which is used as the train’s traction. The long stator is composed of silicon steel sheets. It has the alveolar structure shown in Figure 3. The detection coils of the sensor face the alveolar structure. When a sensor moves along the long stators, it can distinguish the teeth and the slots. By counting the passed alveolar structure, the train’s position is detected [8,9]. Generally, the position sensor has the three functions:

1. *Alveolar counting*: The position sensor counts the passed alveolar structure, and then sends the result to the signal processing unit.
2. *Phase detection*: The detection accuracy can only reach one alveolar structure’s length which can hardly fulfill the requirements of the traction system, so the position sensor needs to give a subdivision. The phase signal between zero degrees to sixty degrees represents the length of one alveolar structure.
3. *Judging direction*: The direction signal is not only the foundation of the sensor’s alveolar counting, but also the precondition of the unit of signal processing to judge the train’s direction.

The signal processing unit is the core component of the speed and position detection system. Each system has one signal processing unit, which is used to receive and handle the sensors’ data. The signal processing unit sends the data processing results to the traction system in terms of the agreement. To some extent, it is the bridge between the traction system and all kinds of sensors. Generally, the signal processing unit has the following main functions:

1. Receiving the sensors’ position signal, speed signal and direction signal;
2. Data filtering and information fusion in accordance with the requirements of the traction system;
3. Communicating with the protocol processing unit. The processed position and direction data is sent to the traction system by the protocol processing unit.

**Figure 4.**
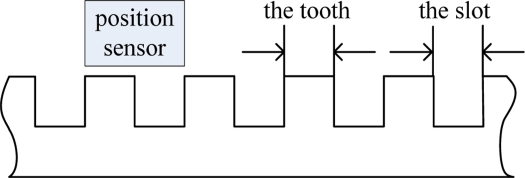
The position sensor and the long stator.

The data that the signal processing unit sends to the traction system includes the following content:

1. Magnetic pole phase (zero degree to three hundred and sixty degrees): The magnetic pole phase signal of is used for the traction, where every sixty degrees represents one alveolar structure. It is calculated by the alveolar counting signal and phase detection.
2. Magnetic pole counting (0 to 65,535): As shown in the Figure 4, each time the train passes six alveolar structures, the magnetic pole counting signal increases by one or decreases by one in terms of the direction.

## 3. Characteristics of the Discrete Tracking-Differentiator Filter

### 3.1. The Linear Discrete Track-Differentiator

The classical differentiator makes use of an inertia element to track the input signal with a time delay. It has the following form [5]:

(1) $$u^{\prime }(t)=\frac{u(t)-u(t-\tau )}{\tau }$$

But the classical differentiator also amplifies the noise, so another approximate form is always used:

(2) $$u^{\prime }(t)\approx \frac{u(t-\tau_{1})-u(t-\tau_{2})}{\tau_{2}-\tau_{1}},0<\tau_{1}<\tau_{2}$$

where, the signals *u*(*t* – *τ*_1_) and *u*(*t* − *τ*_2_) are obtained from the inertia element. Then, the noise amplificatory effect can be reduced. Then there is the transfer function of the differentiator:

(3) $$\begin{array}{l} y=\frac{1}{\tau_{2}-\tau_{1}}(\frac{1}{\tau_{1}s+1}-\frac{1}{\tau_{2}s+1})u\\ \;\;\;\;=\frac{s}{\tau_{1}\tau_{2}s^{2}+(\tau_{1}+\tau_{2})s+1}u \end{array}$$

If the time-delay parameters *τ*_1_ and *τ*_2_ are equal to *ω*, the formula (3) can be turned into the following expression:

(4) $$\frac{Y(s)}{U(s)}=\frac{\omega^{2}s}{s^{2}+2\omega \xi s+\omega^{2}}$$

It can be discretized into a two order equation of state:

(5) $$\{\begin{array}{l} x_{1}(k+1)=x_{1}(k)+h\cdot x_{2}(h)\\ x_{2}(k+1)=x_{2}(k)+h\cdot (-\omega^{2}(x_{1}(k)-u(k))-2\xi \omega x_{2}(k)) \end{array}$$

The formula (5) is the discrete form of the linear track-differentiator. The variable *x*_1_ (*k* + 1) tracks the input signal *u*(*k*).The parameter *ξ* is the damping factor and *ω* is the quickness factor. The bigger the dereferencing of *ω* is, the better *x*_1_ (*k* + 1) approximates *u*(*k*). And then *x*_2_ (*k* + 1) is the approximate value of *u̇*(*k*). However, the linear track-differentiator’s noise immunity is very limited. In order to suppress the noise and track the input signal quickly, this paper adopts a new nonlinear track-differentiator filter to deal with the input signal. The track-differentiator this paper uses is different from Han’s one [10]. The return-to-zero strategy near the switching curve is proposed [11].

### 3.2. Signal Filter Based on the Nonlinear Track-Differentiator

First of all, we consider the problem of a second-order continuous-time system:

(6) $$\left[\begin{array}{c} {\dot{x}}_{1}\\ {\dot{x}}_{2} \end{array}\right]=\left[\begin{array}{cc} 0 & 1\\ 0 & 0 \end{array}\right]\left[\begin{array}{c} x_{1}\\ x_{2} \end{array}\right]+\left[\begin{array}{c} 0\\ 1 \end{array}\right]u$$

The initial state is *X* (*t*_0_) = *X*_0_ and the permissible control domain is |*u*| ≤ *r*. The problem is that how to find the time optimal control to make the system performance index $J=\int_{t_{0}}^{t_{f}}dt$ minimum.

There exists one optimal control strategy. It can make arbitrary point in the phase plane reach the origin using at the most once switching point and can spend the shortest time in doing so. The strategy is called bang-bang control. All of the control switching points compose the switching curve:

(7) $$\Gamma (x_{1},x_{2},r)=x_{1}+\frac{1}{2\cdot r}x_{2}\cdot \left|x_{2}\right|$$

According to the optimal control theory, the optimal solution of the optimal control problem above is as follows:

(8) $$u(t)=\{\begin{array}{l} -r\cdot \mathrm{sign}\;(\Gamma (x_{1},x_{2},r)),\Gamma (x_{1},x_{2},r)\neq 0\\ \text{r}\cdot \mathrm{sign}\;(x_{2}),\Gamma (x_{1},x_{2},r)=0,x_{2}\neq 0 \end{array}$$

If *x*_1_ (*t*) is substituted by *x*_1_ (*t*) − *v*(*t*), then the switching curve is as follows:

(9) $$\Gamma (x_{1},x_{2},r)=x_{1}(t)-v(t)+\frac{1}{2\cdot r}x_{2}(t)\cdot \left|x_{2}(t)\right|$$

The continuous-time track-differentiator is obtained:

(10) $$\{\begin{array}{l} {\dot{x}}_{1}=x_{2}\\ {\dot{x}}_{2}=-r\cdot \mathrm{sign}\left(x_{1}-v(t)+\frac{x_{2}\left|x_{2}\right|}{2r}\right) \end{array}$$

If the arbitrary point *M*(*x*_10_, *x*_20_) is not in the switching curve, *t_A_* is used to represent the time that *M*(*x*_10_, *x*_20_) spends to reach the switching curve. *t_A_* is calculated as follows:

(11) $$t_{A}=s\cdot \frac{x_{2}}{r}+\sqrt{s\cdot \frac{x_{1}}{r}+\frac{1}{2}\frac{x_{2}^{2}}{2\;r^{2}}}$$

If the arbitrary point *M*(*x*_10_, *x*_20_) is in the switching curve, *t_B_* is used to represent the time that *M*(*x*_10_, *x*_20_) spends to reach the origin. *t_B_* is calculated as follows:

(12) $$t_{B}=\frac{\left|x_{2}\right|}{r}$$

In order to get the fastest control function, the step-size unchanged strategy is adopted. The paper [6] proposes the discrete form of nonlinear track-differentiator:

(13) $$\{\begin{array}{l} \left[\begin{array}{c} u(k)\\ b \end{array}\right]=\mathrm{fast}(x_{1}(k)-v(k),c_{1}x_{2}(k),r,c_{0}h)\\ x_{1}(k+1)=x_{1}(k)+h\cdot x_{2}(k)+r\cdot \frac{1}{2}h^{2}\cdot u(k)-\frac{1}{3}b\cdot h^{3}\\ x_{2}(k+1)=x_{2}(k)+r\cdot h\cdot u(k)-\frac{1}{2}b\cdot h^{2},k=0,1,2\cdots . \end{array}$$

where *x*_1_ (0) = *x*_10_, *x*_2_ (0) = *x*_20_.

The term *r* is the quickness factor. It decides the tracking time. *h* is the sampling period. A suitable *h* makes the filtering effect better. If *h* is to too large, the phase of the follow-up signal may suffer from errors. Unless stated otherwise, this paper chooses one millisecond as sampling period. *c*_1_ is the damping factor and *c*_0_ is the filtering factor. The nonlinear function *fast*(*x*_1_, *x*_2_, *r*, *h*) is inferred by the rules as follows:

1. When *M*(*x*_1_ (*k*), *x*_2_ (*k*)) hasn’t reached the switching curve, meanwhile *t_A_* ≥ *h*, it has the following expression:
  (14) $$\{\begin{array}{l} b=0\\ u(k)=-\mathrm{sign}\left(x_{1}(k)+\frac{1}{2\cdot r}x_{2}(k)\cdot \left|x_{2}(k)\right|\right) \end{array}$$
2. When *M* (*x*_1_ (*k*), *x*_2_ (*k*)) hasn’t reached the switching curve, meanwhile *t_A_* < *h*, it has the following expression:
  (15) $$\{\begin{array}{l} b=0\\ u(k)=-\frac{1}{2}+\frac{x_{2}(k)\cdot s}{r\cdot h}+\frac{1}{2}\sqrt{1+\left(\frac{4\cdot x_{2}(k)}{r\cdot h}+\frac{8\cdot x_{1}(k)}{r\cdot h_{2}}\right)\cdot s} \end{array}$$
  where $s=\mathrm{sign}(\Gamma (x_{1},x_{2},r))=\mathrm{sign}\left(x_{1}+\frac{1}{2\cdot r}x_{2}\cdot \left|x_{2}\right|\right)$.
3. When *M* (*x*_1_ (*k*), *x*_2_ (*k*)) hasn’t been in the switching curve, meanwhile *t_B_* ≥ *h*, it has the following expression:
  (16) $$\{\begin{array}{l} b=0\\ u(k)=-\mathrm{sign}(x_{2}(k)) \end{array}$$
4. When *M* (*x*_1_ (*k*), *x*_2_ (*k*)) hasn’t been in the switching curve, meanwhile *t_B_* < *h*, it has the following expression:
  (17) $$\{\begin{array}{l} b=\frac{6\cdot (x_{2}(k)\cdot h+2\cdot x_{2}(k))}{r\cdot h^{3}}\\ u(k)=a+bh \end{array}$$
  where $a=-\frac{2\cdot (2\cdot x_{2}(k)\cdot h+3\cdot x_{1}(k))}{r\cdot h^{2}}$.

In general, the function of fast has the form as this: *fast* (*x*_1_ − *v*(*k*), *x*_2_, *r*, *c*_0_ *h*), which just needs some substitution work for the four rules above.

On the one hand, this track-differentiator can quickly track an input signal and can produce a good differential signal. However the differential signal is not used in this paper. On the other hand, the output signal’s amplitude has almost no attenuation and its time delay is small. Because of the little attenuation of the signal amplitude and linear relations between the time delay and signal frequency, the true value can be separated effectively from signals mixed with noise, so this track-differentiator has the capability to act as an effective filter [11–13]. The relevant proof can been found in [13].

### 3.3. Signal Filter Based on Track-Differentiator Group

**Figure 5.**
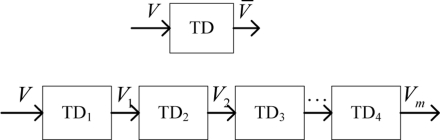
Comparison between track-differentiator and track-differentiator group.

As far as the single track-differentiator, the output signal *V̄* is a kind of function on the filtering factor *c*_0_, damping factor *c*_1_, quickness factor *r* and time-delay factor *τ*. The output signal of track-differentiator has a time delay. The solution is to modulate the correlated parameters, but tracking errors will then increase. The track-differentiator group is a kind of method to relieve the contradiction error and time delay. It is shown as Figure 5. The track-differentiator filter is called TD for short.

where *V*(*t*) is the signal to be tracked. The tracking error is marked as *ξ*(*t*). It is caused by the filtering system and the input signal. Considering the tracking error and time delay, the output of TD is as follows:

(18) $$\bar{V}(t)=V(t-\tau )+\xi (t)$$

With regard to the system with one TD, Taylor expansion of *V*(*t* − *τ*)about t is obtained:

(19) $$V(t-\tau )=V(t)+V^{\prime }(t)(-\tau )+\frac{V^{\prime \prime }{\left(t\right)}^{2}}{2!}{\left(-\tau \right)}^{2}+\frac{V^{\prime \prime \prime }{\left(t-\tau -t\right)}^{3}}{3!}{\left(-\tau \right)}^{3}+R(t,\tau )$$

In the case of a TD of series length m, the output of *TD_i_* is the input of *TD_i_*_+1_, if necessary. The input signal of the track-differentiator group passes by a number of TD, an output with a nice filtering effect will be obtained. So the track-differentiator group may overcome the drawback of one TD’s weak filtering. Meanwhile the tracking error is considered to approach to zero. But the phase compensation comes to be the principal problem. One TD may bring the time delay of *τ*, then a length of m series of TD may result in m times of *τ*. Some sort of phase compensation is needed.

Firstly, the track-differentiator group’s input-output model is given as follows:

(20) $$\{\begin{array}{l} V_{1}(t)=V(t-\tau )\\ V_{2}(t)=V(t-2\tau )\\ V_{3}(t)=V(t-3\tau )\\ V_{4}(t)=V(t-4\tau )\\ \vdots \\ V_{m}(t)=V(t-m\tau ) \end{array}$$

Like in formula (19), Taylor expansion n of *V*(*t* − *τ*) about t is obtained:

(21) $$V(t-i\tau )=V(t)-V(t)•i\tau +\frac{V^{\prime \prime }(t)}{2!}•{\left(i\tau \right)}^{2}-\frac{V^{\prime \prime \prime }(t)}{3!}{\left(i\tau \right)}^{3}+R(t,\tau )$$

If *m* = 4, the high order term is omitted, it has the following form:

(22) $$\left[\begin{array}{c} V_{1}\\ V_{2}\\ V_{3}\\ V_{4} \end{array}\right]=\left[\begin{array}{cccc} 1 & -\tau & \frac{1}{2}\tau^{2} & -\frac{\tau^{3}}{6}\\ 1 & -2\tau & \frac{{\left(2\tau \right)}^{2}}{2} & -\frac{\left(2\tau^{3}\right)}{6}\\ 1 & -3\tau & \frac{{\left(3\tau \right)}^{2}}{2} & -\frac{\left(3\tau^{3}\right)}{6}\\ 1 & -4\tau & \frac{{\left(4\tau \right)}^{2}}{2} & -\frac{\left(4\tau^{3}\right)}{6} \end{array}\right]•\left[\begin{array}{c} V(t)\\ V^{\prime }(t)\\ V^{\prime \prime }(t)\\ V^{\prime \prime \prime }(t) \end{array}\right]$$

To make it further, the parameter of time delay *τ* is drawn out from the matrix of coefficients, and then the matrix equation has the following form:

(23) $$\left[\begin{array}{c} V_{1}\\ V_{2}\\ V_{3}\\ V_{4} \end{array}\right]=\left[\begin{array}{cccc} 1 & -1 & \frac{1}{2} & -\frac{1}{6}\\ 1 & -2 & 2 & -\frac{4}{3}\\ 1 & -3 & \frac{9}{2} & -\frac{9}{2}\\ 1 & -4 & 8 & -\frac{32}{3} \end{array}\right]•\left[\begin{array}{c} V(t)\\ \tau V^{\prime }(t)\\ \tau^{2}V^{\prime \prime }(t)\\ \tau^{3}V^{\prime \prime \prime }(t) \end{array}\right]$$

To get expression which is relative every TD’output, the matrix of coefficients is inverted as follows:

(24) $$\left[\begin{array}{c} V(t)\\ \tau V^{\prime }(t)\\ \tau^{2}V^{\prime \prime }(t)\\ \tau^{3}V^{\prime \prime \prime }(t) \end{array}\right]=\left[\begin{array}{cccc} 4 & -6 & 4 & -1\\ 4.333 & -9.5 & 7 & -1.8333\\ 3 & -8 & 7 & -2\\ 1 & -3 & 3 & -1 \end{array}\right]•\left[\begin{array}{c} V_{1}\\ V_{2}\\ V_{3}\\ V_{4} \end{array}\right]$$

From the matrix equation of (24), the approximation of input signal *V*(*t*) is obtained and it also serves as the output of track-differentiator group:

(25) $$\bar{V}(t)=4V_{1}(t)-6V_{2}(t)+4V_{3}(t)-V_{4}(t)$$

Considering the generalized case, the tracking signal with phase compensation is as follows:

(26) $${\bar{V}}_{m}=\sum_{i=1}^{m}\alpha_{mi}V_{i}$$

When the variable *m* is assigned different value, the coefficient *p_m_* of *α_mi_* makes a difference. Theoretically, as the value of *m* increases, the calculations needed will be considerable. In actual use, the order of the track-differentiator should be chosen based on the system’s requirement and computational capabilities.

## 4. Signal Process for System of Position and Speed Detection

### 4.1. Description of the Sensor’s Abnormal Signal

**Figure 6.**
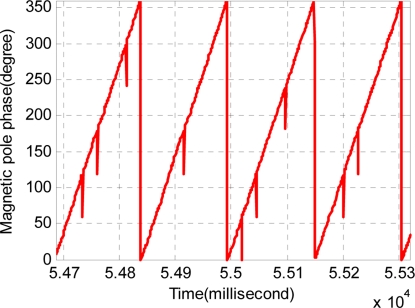
The magnetic pole phase because of asynchronization.

In theory, the signal of phase detection increases or decreases in terms of the train’s running direction between zero degrees to sixty degrees. In the forward running condition, as the phase detection signal changes from sixty degrees to zero, the alveolar counting signal increases by one. In the backward condition, as the phase detection signal changes from zero degree to sixty, the alveolar counting signal decreases by one. But in the real circuit processing, the sine wave obtained by synchronous demodulation is sent to a different post-processing circuit, and then the change of phase signal and alveolar counting signal are not synchronized. If the signal processing unit simply integrates them, there will be a mutation as shown in Figure 6. The amplitude is about sixty degrees.

As the position sensor moves along the long stators, the inductance of its coil will change. In short, the position sensor can detect the change of different metal’s inductance. Meanwhile the position sensor is installed at the bottom of the train, so its detection gap is changeable with the suspension height. Then the fluctuating gap may also make the inductance change, which makes the vertical and horizontal changes coupled. In other words, the sensor can hardly distinguish between vertical and horizontal changes, so the linearity of its position detection will be affected.

**Figure 7.**
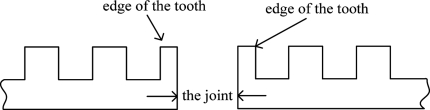
The joint of long stators.

**Figure 8.**
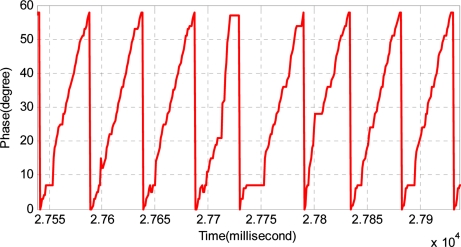
The aberrant signal affected by joint.

For prevention of thermal expansion and contraction and also for convenience when laying out the long stators, the long stator track is composed of numerous stators, so there are many joints. Usually, the length of the joint is seventy to eighty millimeters. This is shown in Figure 7. There is nothing except track cable in the joint. The existence of joints breaks the continuity and the periodicity of track inductance and increases the difficulty of detection. As the position sensor is passing the track joint, the position signal will be more or less aberrant. Figure 8 shows the affected phase.

### 4.2. Pre-Treatment of the Position Sensors

The signal processing unit receives the phase and alveolar counting signal to locate the train. Then the train’s position is equal to the phase signal plus the alveolar counting signal. To avoid the low-pass effect of the track-differentiator, the signal processing unit needs to pre-treat the signals.

The frequency spectrum of the position signal must be within the track-differentiator’s frequency band, so the high-frequency noise can be filtered. Considering the relation of phase and alveolar counting, it can be easily found that the phase is a subdivision of alveolar counting. The phase signal can be treated as the low bit while the alveolar counting is the high bit, so the saw-tooth wave is turned into a ramp wave. Then they can be integrated as follows:

Firstly, considering the range of phase signal *x*_60_, it can be normalized into the range of zero to ten. The method is as follows:

(27) $$x_{10}=\frac{x_{60}}{m}•10=\frac{x_{60}}{60}•10$$

**Figure 9.**
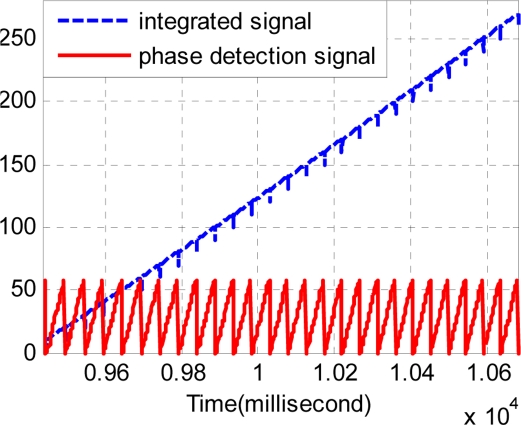
The phase detection and integrated signal in the forward direction.

**Figure 10.**
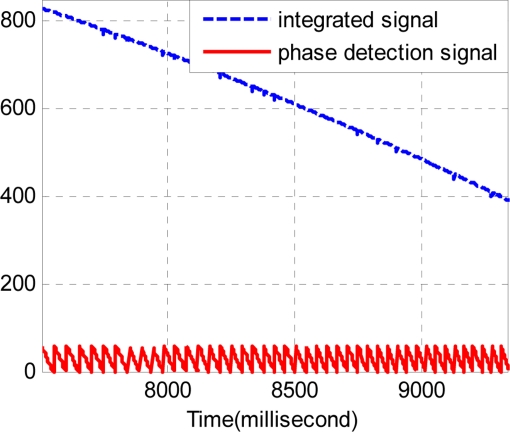
The phase detection and integrated signal in the backward direction.

Secondly, the normalized result *p* can be integrated with the signal of alveolar counting *y*. It is marked as formula (28). Especially under uniform velocity conditions, the integrated result will be similar to a ramp wave. Figure 9 shows the forward phase signal and the integrated result. Figure 10 shows the backward phase signal and the integrated result. Note that the integrated signal is dimensionless.

(28) $$p=x_{10}+y•10$$

### 4.3. Application of the Track-Differentiator in Speed and Position Detection System

**Figure 11.**
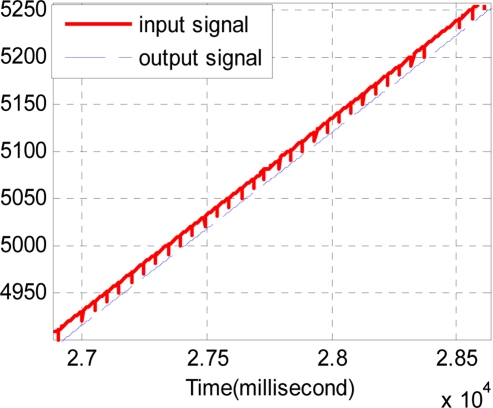
Comparison of the input and the output signal.

**Figure 12.**
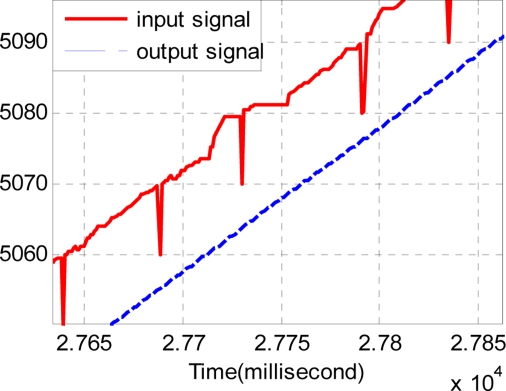
Partial enlarged drawing of Figure 11.

The sample period of the discrete track-differentiator is one millisecond, and the quickness factor *r* is 1,500. Figures 11 and 12 show the comparison of the input signal and the output signal. It can be seen that the track-differentiator can effectively smooth the input signal and remove the impulse interference.

From comparison of the input curve and the following curve in Figure 12, the filtering results in the time delay just as the analysis of Section 3.3. Even though the filter produces a nice smooth effect, the output signal cannot be used because of the time delay.

**Figure 13.**
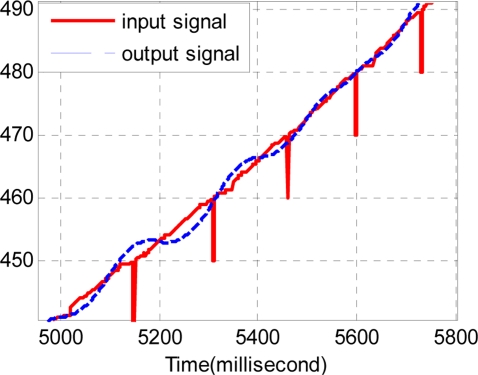
Comparison of the input signal and the output signal.

Meanwhile the damping factor *c*_1_ can be decreased to make time delay smaller. But if *c*_1_ gets smaller, the filtering effect will get worse. It is shown in Figure 13 that furthermore the quickness factor *r* and the filtering factor *c*_0_ are modulated, the conflict of filtering and time delay still cannot be resolved.

### 4.4. Application of the Track-Differentiator Group in Speed and Position Detection System

**Figure 14.**
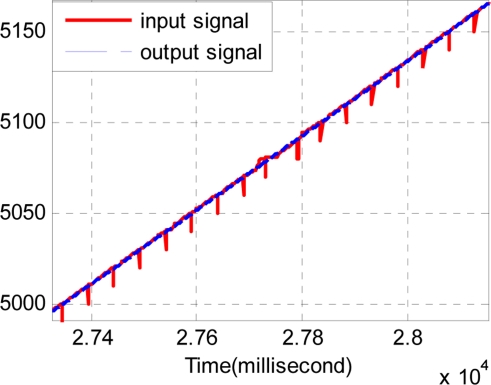
Comparison of the input signal and the output signal.

**Figure 15.**
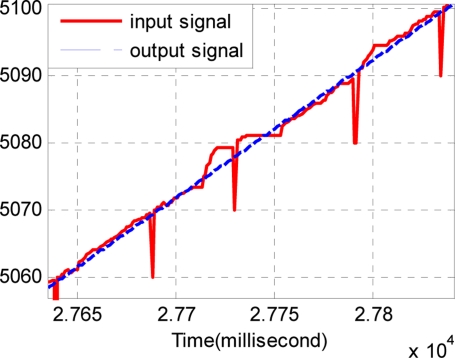
Partial enlarged drawing of Figure 14.

Considering the same noisy conditions of Section 4.3, four series of TD are used. Each TD has the same quickness factor, filter factor and damping ing factor, where *r* = 1500, *c*_0_ = 2, *c*_1_ = 1.5. The output of the track-differentiator group is shown in Figures 14 and 15. It can be seen that the output signal can follow the input signal nearly without time delay. Also Figure 15 shows that the pulse noise has been eliminated and the aberrant signal has been repaired.

**Figure 16.**
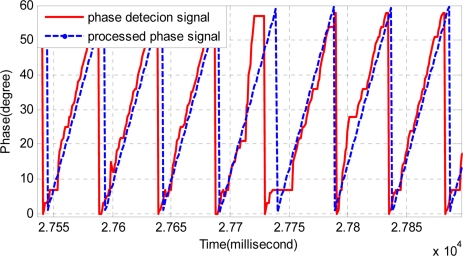
Comparison of the phase detection and processed phase signal.

According to the introduction of Section 2, the signal processing unit needs to send the magnetic pole phase to the traction system, so it is recommended to recover the integrated signal to the phase signal and alveolar counting signal. Because the recovered phase signal is processed by the track-differentiator group, it is smoother and has less noise. This is shown in Figure 16.

## 5. Conclusions

This paper analyses the reason that the phase detection signal and alveolar counting signal of the position sensor are unsynchronized. Aiming at eliminating the noise and overcoming the effect of the long stator joints, a new non-linear discrete track-differentiator is proposed. Meanwhile the track-differentiator group is also designed and analyzed. Especially, a four series TD is used in the speed and position detection system systems of a permanent magnet electric suspension train. In the train’s actual operation, it has played a good effect.

## Figures and Tables

**Table 1.** Coefficients *α_mi_*.

*m*	*α*_*m*1_	*α*_*m*2_	*α*_*m*3_	*α*_*m*4_	*α*_*m*5_	*α*_*m*6_
2	2	−1				
3	3	−3	1			
4	4	−6	4	−1		
5	5	−10	10	−5	1	
6	6	−15	20	−15	6	−1
